# Variation of the Antioxidative Defense in *Elaeis guineensis* Jacq. Facing Bud Rot Disease in the Coastal Area of Ecuador

**DOI:** 10.3390/molecules27217314

**Published:** 2022-10-27

**Authors:** Raluca A. Mihai, Galo M. Canchignia Guacollantes, Sebastián A. Villacrés Mesias, Larisa I. Florescu, Rodica D. Catana

**Affiliations:** 1 CICTE, Department of Life Science and Agriculture, Universidad de las Fuerzas Armadas—ESPE, Av. General Rumiñahui s/n y Ambato, Sangolquí 171103, Ecuador; 2 Pasteurizadora Quito, Pedro Pinto Guzmán 610 y Av. Napo, Quito 170604, Ecuador; 3 Institute of Biology Bucharest, Romanian Academy, 060031 Bucharest, Romania

**Keywords:** antioxidant activity, bud rot disease, defense mechanism, phenolic compounds, palm oil

## Abstract

*Elaeis guineensis* Jacq. has gained a reputation in the food industry as an incredible crop capable of supplying the world’s largest edible oil production. In Ecuador, an important oil palm-producing country, this crop is affected in a high percentage by the bud rot disease, which is responsible for palm death. The main objective of the investigation was dedicated to understanding the palm defense mechanism facing bud rot disease, translated in the induction of reactive oxygen species, activation of defensive machinery comprising enzymatic and non-enzymatic antioxidative components, secondary metabolites, carotenoids accumulation in the palm during all stages of disease infection. For this, a survey was conducted in different oil palm plantations in the Esmeraldas province, one of the most representative for its highest incidence of bud rot disease. The survey completed DPPH, FRAP, ABTS, and other spectrophotometric analyses to underline the biochemical, biological, and physiological palm response front of bud rot incidence. The palm defense strategy in each disease stage could be represented by the phenolic compound’s involvement, an increment of antioxidant activity, and the high enzymatic activity of phenylalanine ammonia-lyase (PAL). The results of the investigation made understandable the palm defense strategy front of this disease, respectively, the antioxidative defense and the palm secondary compounds involved.

## 1. Introduction

Palm oil (*Elaeis guineensis* Jacq.) is a perennial crop with a more than 25-year life cycle, originally from the West Coast of Africa. It grows at ± 10° latitude of the equator (in Africa, South East Asia, and South and Central America) in the wild, semiwild, and cultivated parts of the tropics [1]. It represents the world’s most important species of stem-less Arecaceae family, tree-like monocot plants, which are very important to humans and biodiversity, especially in the tropics [2]. This crop gains its reputation in the food industry with one of the most versatile oils for food applications and one of the richest dietary sources of pro-vitamin A and vitamin E due to their balanced fatty acid composition. Palm oil is a crop leader source of vegetable oil with an exceptionally low land footprint compared to annual oilseed crops, such as soybean, rapeseed, and sunflower. Globally, palm oil supplies 40% of the world’s traded vegetable oil demand on just under 6% of the land used to produce all vegetable oils. Oil palm is crucial to the economies of many countries, especially Indonesia and Malaysia [3], and is widely cultivated in plantations across the humid tropics of Asia, Africa, and the Americas.

Ecuador is an important producer of crude palm oil in Latin America, but with yields per hectare lower than Colombia and Costa Rica. It is the seventh-largest producer worldwide, in a market dominated by Indonesia, Malaysia, and Thailand. In Ecuador, the oil palm has been described as a growing, stable, and successful productive chain until the situation changed when the oil palm crop started to face many challenges in the 2020s. This negative situation for Ecuador was translated into a reduction and loss of export earnings, a decrease in the economy, and a high incidence of poverty in the palm oil-producing areas due to massive job losses. The cause for all these was provoked by an increasing incidence of existing diseases, such as bud rot, a catastrophic one, capable of affecting more than 57% of the plantations at the national level [4].

Bud rot type disease was reported for the first time in the 1920s on oil palm plantations in Suriname, followed by another incidence in Panama, with two forms: a lethal (in Ecuador, Brazil, certain zones of Colombia, and Suriname) and a non-lethal one (found mainly in the Colombian Llanos), that only inhibits the growth of the palm for some period and has a good to higher recovery rate [5].

The bud rot type disease symptoms start with chlorosis of the youngest leaves, followed by necrosis causing a collapse of the spear leaf and plant death [6]. The first symptom consists of a brown lesion at the upper region of the spear. The following spears present more severe symptoms when the “Bud Rot” advances into the vascular tissues and the primordial foliage [7]. The bud rot disease has become the main cause of deterioration and loss of plantations in Ecuador with a high incidence in the coastal areas, especially in the Esmeraldas province. Weather parameters (temperature and relative humidity) of this area of the country are naturally favorable for the high incidence and spread of bud rot disease.

The history of the etiology of this disease at the international level has been approached from two perspectives: biotic and abiotic; with more information on the biotic one, which presents an abundance of hypotheses, arguments, contradictions, and conclusions for the responsibility attributable to the organisms involved in this pathogenic process, such as *Erwinia* sp. for bacteria [8]; *Thielaviopsis* and *Fusarium* for fungi [9]; and *Pythium* and *Phytophthora* for Oomycete [10]. It is known that, in general, pathogens can disturb physiological and metabolic processes and pathways in plants resulting in loss of yield and quality plants [11]. During pathogen infection, plant tissues accumulate a high level of reactive oxygen species (ROS) at the level of the attack site through the oxidative burst phenomenon [12], causing photo-oxidative damage to biomolecules and to the internal cellular structures [13,14]. In plants, ROS, in both forms (the non-radical and free radical forms) plays the role of secondary messenger for numerous signaling reactions and induces biochemical changes associated with stress signaling that activate their defense pathways, leading to the death zone of the host cell, preventing, in this way, the spread of biotrophic pathogens [14]. The induced defense mechanism includes various non-enzymatic components comprising phenolic compounds, flavonoids, and enzymes for phenol metabolism, such as phenylalanine ammonia-lyase (PAL) [15], and it is also reported that carotenoids serve as a defense mechanism for the detoxification of several types of ROS.

This manuscript emphasized the defense mechanism of palm oil facing bud rot in each stage of disease involving the synthesis of palm secondary metabolites implicated in neutralizing the negative effects of biotic stress translated also into the rapid production and accumulation of ROS.

## 2. Results

### 2.1. Total Polyphenolic Content and Total Flavonoid Content

The higher amount of total polyphenolic content was registered in the case of bud rot disease stage I (E I), followed by disease stage II (E II), and Stage III (E III) (Table 1).

**Figure 1:**
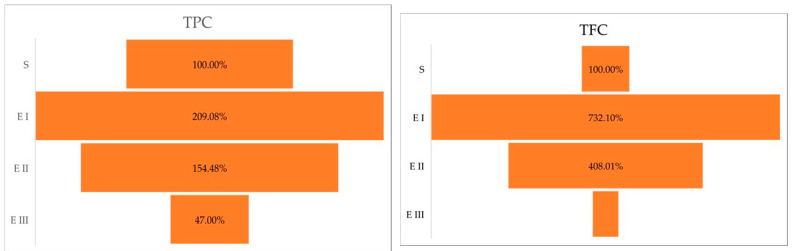
Total polyphenolic and flavonoid content (%) in different stages of the disease. Legend: S—healthy palm oil; E I—stage I of bud rot disease infection in oil palm; E II—stage II of bud rot disease infection in oil palm; E III—stage III of bud rot disease infection in oil palm; TPC—total phenolic content; TFC—total flavonoid content.

The Duncan post hoc test of the ANOVA (*p* < 0.0001) showed the significance of the differences between healthy plants vs. disease stage I (*p* < 0.0001); healthy plants vs. disease stage II (*p* = 0.0009); and healthy plants vs. disease stage III (*p* = 0.001). Compared with the healthy plants (S), the amount of total polyphenol content was higher in stages I and II. In stage III, the amount of total polyphenol content was lower than in healthy plants. The same trend was observed in the case of total flavonoid content, the higher amount was registered in the case of disease stage I followed by Stages II and III (Table 1). The Duncan post hoc test of ANOVA (*p* < 0.0001) has caught differences between the total flavonoid content of healthy plants vs. disease stage I (*p* < 0.0001); healthy plants vs. disease stage II (*p* = 0.0002); and no significant variance between healthy plants vs. disease stage III (*p* = 0.2384). A percentual comparison (Figure 1) showed that in stage I, plant cells accumulate 209.08% TPC and 732.10% TFC higher than the healthy plants.

### 2.2. Antioxidant Activity

The antioxidant properties of the oil palm in different stages of bud rot disease infection emphasize the differences between all three methods used (Table 2).

**Figure 2:**
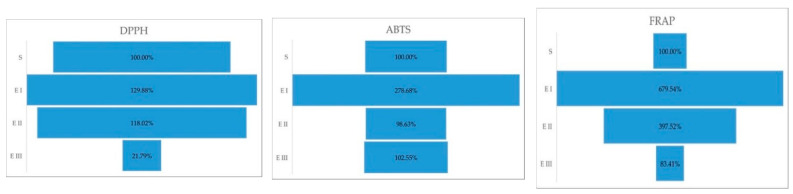
Antioxidant capacity (ABTS, DPPH, FRAP) expressed in percent, during the different stages of the disease. Legend: S—healthy palm oil; E I—stage I of bud rot disease infection in oil palm; E II—stage II of bud rot disease infection in oil palm; E III—stage III of bud rot disease infection in oil palm.

In DPPH activity, the one-way ANOVA (*p* = 0.0009) emphasized by the Duncan test showed significant differences between all three stages (*p* = 0.0017). In the case of ABTS, the ANOVA (*p* < 0.0001) test presented the highest significant differences between healthy plants vs. disease stage I (*p* < 0.0001) followed by healthy plants vs. disease stage III (*p* = 0.0311). No difference was found between healthy plants vs. disease stage II (*p* = 0.5113). For FRAP, ANOVA (*p* < 0.0001) with the post hoc Duncan test showed significant changes between healthy plants vs. disease stage I (*p* < 0.0001); healthy plants vs. disease stage II (*p* < 0.0001); and no significant variance between healthy plants vs. disease stage III (*p* = 0.0635). The percentual comparison showed that in stage I, plant cells have higher antioxidant capacity than the healthy plants; more than 2.5× higher (ABTS) and more than 6.5× than healthy plants (Figure 2).

To test potential influences between secondary metabolites (total phenolic content and total flavonoid content) and antioxidant activity (FRAP, DPPH, ABTS), Pearson correlations were applied. A significant result was identified only between TPC and FRAP (R = −0.99, *p* = 0.0342) in stage III of the disease. Positive correlations were identified between total phenolic content and antioxidant activity identified through FRAP assay (R = 0.955; *p* = 0.04) and between total flavonoid content and FRAP (R = 0.992; *p* = 0.0008).

### 2.3. Chlorophyll a, b and Total Carotenoids Content

**Figure 3:**
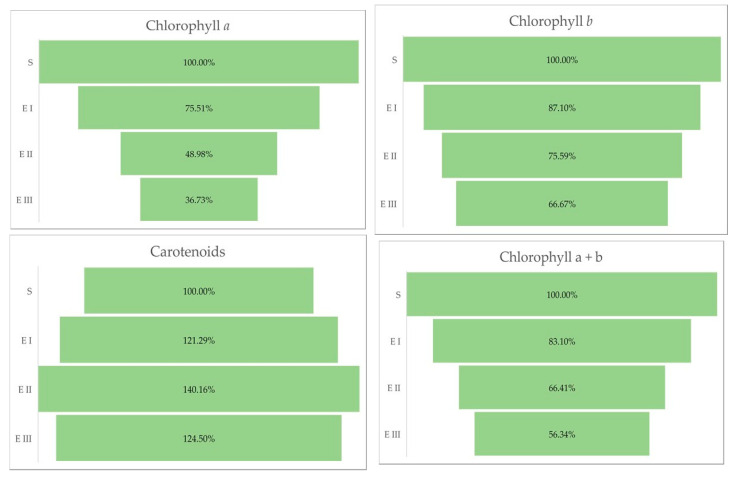

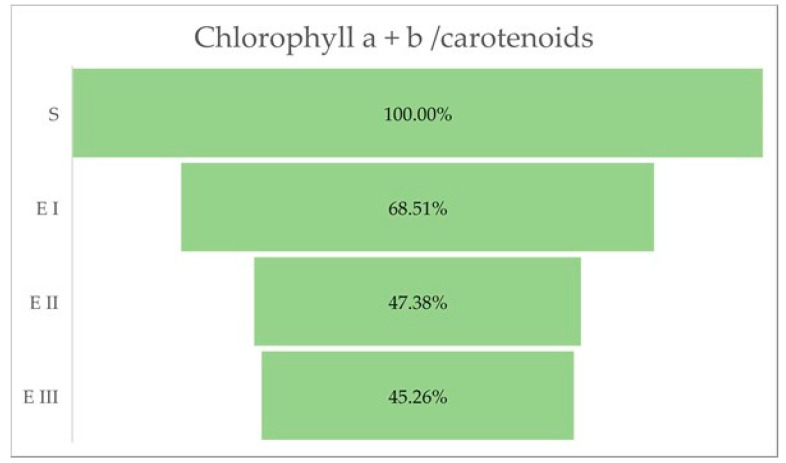
Chlorophylls a, b, and carotenoids (%) in different stages of the disease. Legend: S—healthy palm oil; E I—stage I of bud rot disease infection in oil palm; E II—stage II of bud rot disease infection in oil palm; E III—stage III of bud rot disease infection in oil palm.

The quantity of chlorophylls (a and b) was lower in the case of all bud rot disease infection stages in oil palms compared with healthy plants. The same trend was observed for the chlorophyll a + b and the chlorophyll a + b/carotenoids ratio. The all-disease stages of infection were characterized by a higher content of carotenoids compared with healthy plants (Table 3 and Figure 3).

### 2.4. PAL Activity

**Figure 4:**
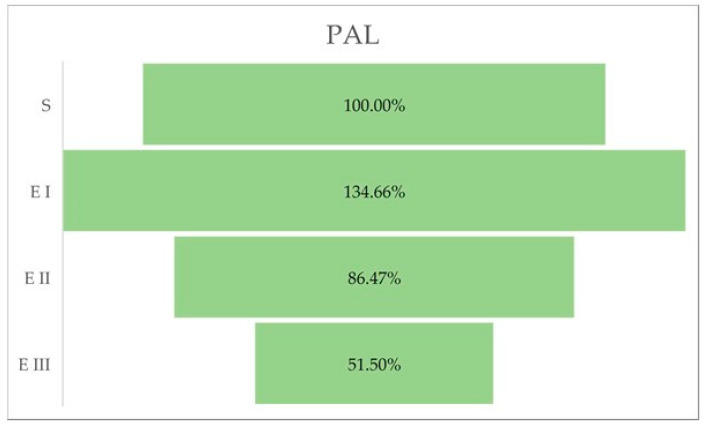
PAL activity (%) in different stages of the disease. Legend: S—healthy palm oil; E I—stage I of bud rot disease infection in oil palm; E II—stage II of bud rot disease infection in oil palm; E III—stage III of bud rot disease infection in oil palm.

The activity of phenylalanine ammonia-lyase (PAL) was higher in stage I of the bud rot disease infection compared with healthy oil palm plants. In the case of the other stages (II and III), PAL activity was lower than in stage I and also lower than in the healthy palms (Table 4, Figure 4).

## 3. Discussion

### 3.1. Total Polyphenol and Total Flavonoid Content Determination

There are many findings about these secondary metabolites focused on plant defense mechanisms against pathogens, including bacteria, fungi, and viruses, and major abiotic stresses, such as nutrition, drought, salinity, and UV. Several phenolics (simple and complex) accumulate in plant tissues and act as phytoalexins, phytoanticipins, and nematicides against soil-borne pathogens and phytophagous insects [16]. When pathogens attack, phenolic compounds are produced by plants, as part of the active defense response [17]. Cherif et al. [18] reported that the early and rapid phenolic accumulation at the site of pathogen infection resulted in isolation and limited the progression of pathogens. This can be the explanation for the increased synthesis of phenolics in the first stage of bud rot disease infection in palm oil, as a natural reaction of palm oil defense against pathogen attack. A decrease of phenolic amount proportionally with the increase of the disease intensity can be due to the loss of the palm’s ability to fight against the agents of the bud rot disease due to the high number of pathogens. The phenolic compounds have been used to serve as alternatives to the chemical control of pathogens of crops, possibly also for palm oil in the future.

Flavonoids (the free state and glycosides) represent the largest group of natural phenolic compounds, occurring in different plant parts. Due to numerous evidence of the biological activities of phenolic compounds, the flavonoids were found to have many biological activities (such as antimicrobial, mitochondrial adhesion inhibition, antiulcer, antiarthritic, antiangiogenic, anticancer, protein kinase inhibition, etc.) [19]. The major roles of flavonoids include the modulation of ROS in plant tissues, being one of the secondary ROS scavenging systems [20]. Agati et al. [21] reported that they are important in scavenging singlet oxygen (1O_2_) and mitigating the destruction that happened to the outer envelope of the chloroplast membrane. Biotic stresses (pathogen attack and herbivore), which represent a potent source of ROS generation, and oxidative stress caused by these free radicals are alleviated by these secondary metabolites [22,23]. This could explain the accumulation of the flavonoids in the first stage of the disease infection as a reaction to protect the palm oil against biotic stress caused by pathogen attack, the trend being similar to the phenolics.

### 3.2. Antioxidant Activity

The ROS production (such as hydrogen peroxide (H_2_O_2_), superoxide anion radical (O2•−), singlet oxygen (1O_2_), and hydroxyl radical (•OH)) represents a common consequence in the cell under biotic or abiotic stress, which may lead to extreme oxidative loss to plant tissues [24]. When plants are attacked by pathogens, they respond by activating a variety of defense mechanisms, including the rapid production and accumulation of these ROS that cause plant cell and pathogen death. ROS detoxifications are carried out when plant cells, enzymes, and redox metabolites function synergistically to protect themselves from adverse effects. The induced defense is facilitated via defensive enzymes along with secondary metabolites (phenols and condensed tannins) [24]. The phenolic compound has redox properties, which allow them to exhibit antioxidants and antimicrobial properties, which help the plant escape pathogenic infections as well as protect the major tissues from the toxic effect of reactive oxygen species [25]. This free radical scavenging ability is facilitated by their hydroxyl groups; the total phenolic concentration could be used as a basis for rapid screening of antioxidant activity. Plant flavonoids have antioxidant activity in vitro and also act as antioxidants in vivo [26].

The present research investigated the antioxidant properties of palm oil through ABTS, DPPH, and FRAP assay at each stage of bud rot disease development, as a defense mechanism caused by the accumulation of ROS under the pathogenic influence. The results indicated a high antioxidant capacity of palm oil in the first stage of infection necessary to inactivate the high synthesis of ROS produced due to pathogen attack. Furthermore, the antioxidant capacity coincides with the highest levels of phenolics and flavonoids—strong antioxidant compounds synthesized as a consequence of the initial stage of the disease.

### 3.3. PAL Activity

PAL is an extensively studied enzyme implicated in the metabolism of phenylpropanoid and aids in synthesizing several secondary metabolites, which include phenols (coumarins, flavonoids, lignins), phenolic derivatives, and lignin [27]. The PAL activity increases in infection [28]. Kaur et al. [29] showed that the PAL activity was increased in the leaves of resistant cultivars of barley genotypes infected with spot blotch pathogen *B. sorokiniana*. The resistant reaction is determined by the defense of the cell walls through lignin intensification and by the accumulation of phenolic compounds around the cell wall [30]. Nicholson and Hammerschmidt [31] showed that the expression of PAL activity and accumulation of phenolic compounds at the infection site has been linked to the resistance mechanism. The total phenolic content has been correlated with host resistance to numerous diseases, e.g., Karnal bunt [32] and *Alternaria* blight [33], in wheat. PAL activity is affected by numerous factors (light, temperature, growth regulators, inhibitors of RNA/protein synthesis, wounding, and mineral nutrition). Increased levels of PAL and flavonoid compounds have been demonstrated in many tissues [34]. Our results indicate the highest PAL activity in stage I of the disease, a fact that suggests that the oil palm defense mechanism involves the activation of PAL and is represented by the synthesis of phenols and flavonoids, where PAL is a key enzyme of the phenylpropanoid pathway from which phenolic metabolites are synthesized, and so the spread of the pathogen is reduced.

### 3.4. Chlorophyll a, b and Total Carotenoids

Carotenoids are yellow, red, and orange color pigments, responsible for both pigmentation properties and for the ability to interact with free radicals and singlet oxygen, a property that makes them responsible for being a powerful antioxidant in the plant system [35]. Carotenoids show their anti-oxidative potential by safeguarding the photosynthetic system by exciting chlorophyll [36].

The aberrant changes caused by bud rot disease, which can be observed at the leaves level, are presented by variations in chlorophyll, a decreased level of chlorophyll pigments, which can also trigger a reduced efficiency of photosynthesis. Therefore, the chloroplasts can be one of the essential organelles that integrate the disease signals and transmit the pre-defense signals for amplification based on ROS accumulation [37]. The greenness of plants may be indicated by the ratio of chlorophylls (a and b) to total carotenoids. The ratios normally lie between 4.2 and 5 in sun leaves and sun-exposed plants, and between 5.5 and 7.0 in shade-exposed plants. Lower values for the ratio are an indicator of senescence, stress, and damage to the plant and the photosynthetic apparatus, which is expressed by a faster breakdown of chlorophyll than carotenoids. In our experiment could be observed a continuous decrease in the ratio, from normal values in the healthy palm to very low values in the third stage of the disease, indicating a high affectation of the palm by stress factor almost until reaching the dead stage.

## 4. Materials and Methods

### 4.1. Materials

A Milli-Q water purification system (Millipore Corp., Bedford, MA, USA) was used for ultrapure water. The 2,2-azinobis-3-ethyl-benzothiazoline-6-sulfonic acid (ABTS), 1,1-diphenyl-2-picrylhydrazyl (DPPH), 2,4,6-tris(2-pyridyl)-(S)-triazine (TPTZ), 6-hydroxy-2,5,7,8-tetramethylchroman-2-carboxylic acid (TROLOX), Folin–Ciocalteau’s reagent FeCl_3_·6H_2_O, FeSO_4_·7H_2_O, and potassium persulfate, cinnamic acid, mercaptoethanol, L-phenylalanine, borate buffer, and trifluoroacetic acid were purchased from Sigma Aldrich (St. Louis, MO, USA), and sodium carbonate from JT Baker (Phillipsburg, NJ, USA). Ethanol and acetone were of HPLC grade and other reagents were of analytical grade.

### 4.2. Plant Material and Sample Collection

The study was conducted in three plantations, located in the Esmeraldas province of the coastal area of Ecuador, at an altitude of 223 m.a.s.l at the GPS coordinates 0°19′48″ N, 79°28′48″ W, Latitude: 0.33, Longitude: −79.48. The climatologic conditions are represented by 23–32 °C average temperature, 85−91% relative humidity, 2.000–3.206 mm annual rainfall, and tropical rainforest agro-ecological conditions. Plots with a high incidence of bud rot disease (incidence > 60%) have been selected, with more than 4-year-old planting material of *E. guineensis* Jacq. Leaflets (from three plants) are collected in the morning hours, from the middle part of leaf number 17 in adult plants. These samples were taken in July, coinciding with times of low rainfall, and not close to fertilization to reduce the variability of the results. The harvested leaflets of each sample were placed in cooler bags and transported directly to the CICTE laboratory of the Universidad de Las Fuerzas Armadas for further analysis.

### 4.3. Palm Extraction

The foliar palm material was washed in water to remove impurities, dried in a circulating-air oven (37 ± 2 °C), and powdered in a mill. The milled palm leaves (5 g) were extracted in 25 mL ethanol (*v*/*v*) for 24 h at 4 °C in obscurity. Next, the extractive solution was filtered, concentrated in a rotavapor under reduced pressure, lyophilized (Martin Christ Alpha 1-4) to yield a crude extract (CE, 272 g), and stored at −20 °C for further analysis.

### 4.4. Determination of the Total Phenolic Content and Total Flavonoid Content

The *total phenolic content* of the extracts was determined using Folin–Ciocalteau assay [38] with few modifications. An aliquot of lyophilized palm leaves extract dissolved in solvent extraction was diluted to 5 mL of Milli-Q water and was added to the 1.5 mL Folin–Ciocalteau reagent, and after 5 min of reaction at room temperature (25 °C), 2 mL of a 100 g/L solution of Na_2_CO_3_ was added. The solution was left for 30 min and its absorbance was measured with a UV–VIS spectrophotometer at 750 nm against a blank without extract. A gallic acid (GA) calibration curve was obtained using standard solutions within the range of 0–250 mg/L. The results are expressed as mg GA equivalent/g of palm leaves (mg GAE/g DW). The obtained equation was expressed y = 0.0112 x + 0.1759, correlation coefficient of the calibration curve was 0.9794 (DL = 2.547 mg/L, QL = 8.4915 mg/L, n = 8, *p* = 2.734 × 106).

The *total flavonoid content* of the crude extract was determined by the aluminum chloride colorimetric method described by Dowd [39]. An aliquot of 1 mL of palm leaves extract solution (25–200 µg/mL) was mixed with 0.3 mL of 10% (*v*/*v*) AlCl_3_ solution in methanol, 0.2 mL (1 M) potassium acetate, and 5.6 mL distilled water. The mix was incubated for 10 min at room temperature, and after the absorbance was measured at 430 nm. The calibration curve was obtained using quercetin (QE) as the standard in the range of 0–1500 mg/L. The calibration curve had a correlation coefficient of 0.9935, with a DL = 1.0412 mg/L and QL = 3.4706 mg/L. The obtained equation was y = 1.4566 x + 0.0265, n = 8, *p* = 8.436 × 108. The results are expressed as mg QE equivalents/g palm leaves weight.

### 4.5. Antioxidant Activity

#### 4.5.1. DPPH Radical Scavenging Assay

The DPPH• radical scavenging activity was determined using the method presented by Simirgiotis et al. [40]. Briefly, 50 µL of processed EtOH extract was added to 2 mL of fresh 0.2 mM solution of DPPH in ethanol and allowed to react at room temperature in the dark. The absorbance was measured at 517 nm. The DPPH scavenging ability as a percentage was calculated as the difference between the absorbance of the control (A control) and the ratio between the absorbance of the sample (A sample) and the control (A control) × 100. Afterward, a curve of % DPPH bleaching activity versus concentration was plotted and IC_50_ values were calculated. IC_50_ denotes the concentration of sample required to scavenge 50% of the DPPH free radicals. The calibration curve was determined using TROLOX standard solutions ranging from 0 to 0.625 mM, obtaining an equation of y = 158.07 x − 1.6766, with a correlation factor of R^2^ = 0.9955 (DL = 0.00017694 mM; QL= 0.0005898 mM, n = 7, *p* = 1.4341 × 106).

#### 4.5.2. Ferric Reducing Antioxidant Power (FRAP) Assay

The FRAP assay was conducted according to the method reported by Benzie and Strain, [38] with few modifications. FRAP reagent was prepared freshly by mixing three solutions 1:1 acetate buffer (300 mM, pH 3.6), a solution of 10 mM TPTZ in 40 mM HCl, and 20 mM FeCl_3_ at 10:1:1 (*v*/*v*/*v*). To complete the reaction, the reagent was kept in darkness for 30 min. The sample was incubated for 30 min in the dark at 37 °C with 2 mL of the FRAP solution (prepared by mixing 25 mL acetate buffer, 5 mL TPTZ solution, and 10 mL FeCl_3_·6H_2_O solution). The increase in absorbance of the reaction mixture was measured for each sample on a UV–VIS spectrophotometer at 593 nm. The calibration curve was obtained at preparing standards of FeSO_4_·H_2_O solutions within the range of 0–2 mM. The equation was y = 1.0583 x − 0.199 (R^2^ = 0.9193, DL= 0.0510 mM, QL = 0.17002 mM, n = 6, *p* = 0.002509645).

#### 4.5.3. ABTS Radical Scavenging Assay

The ABTS radical scavenging assay was carried out according to the method reported by Loizzo et al. [41]. The ABTS radical cation (ABTS+) was generated by mixing the following solutions: 7.0 mM ABTS solution in H_2_O (Solution a) and 2.45 mM potassium persulfate (K_2_S_2_O_8_) solution in H_2_O (Solution b) in a ratio of 1:1 (*v*/*v*), and kept for 16 h at room temperature and in darkness to complete the reaction. After, this solution was diluted with 80% methanol to get the ABTS working solution to an absorbance of 0.700 ± 0.005 at 734 nm. For the assay, a total of 100 µL of appropriately diluted samples were added to 2 mL of ABTS solution and the absorbance was recorded at 734 nm after 1 min of incubation at room temperature. As blank (control), an equal amount of ethanol (100 μL) was used. The samples were vortexed for 1 min. After 6 min of incubation, the decrease in absorbance of each sample was measured against ethanol as blank on UV–VIS spectrophotometer at 734 nm. The percentage of ABTS inhibition was calculated using the formula:

$$\mathrm{ABTS}\;\mathrm{inhibition}\;\left(\%\right)=\frac{A\;\mathrm{Control}-A\;\mathrm{Sample}}{A\;\mathrm{Control}}\times 100$$

The results were reported as IC50 values, a lower IC50 value represents a stronger ABTS scavenging capacity. Standard solutions of TROLOX were prepared within the range of 0–2.5 mM for the calculation of a calibration curve. The obtained equation was y = 31.995 x + 3.9568 with a correlation factor of R^2^ = 0.9697, (DL = 0.001135 mM, QL = 0.003786 mM; n = 8, *p* = 8.81872 × 106).

### 4.6. Quantitative Determination of Chlorophylls a and b and Carotenoids

Palm extracts prepared with 80% acetone, within the range of the appropriate concentration (1.0 to 4.0 mg/mL) were analyzed in a UV–VIS spectrophotometer at 470, 653, and 666 nm. The concentrations of carotenoids and chlorophylls a and b were determined according to the equations reported by Lichtenthaler and Wellburn [42] as follows:

Total carotenoids (mg/L) = 1000 Abs_470_ − 2.860 Ca − 129.2 Cb/245

Chlorophyll a (mg/L) = 15.65 Abs_666_ − 7.340 Abs_653_

Chlorophyll b (mg/L) = 27.05 Abs_653_ − 11.21 Abs_666_

### 4.7. Assay of PAL Activity

PAL activity in the partially purified enzyme extracts obtained through the method of Lister and Lancaster [43] was assayed by an adaptation of the McCallum and Walker method. The assay mixture consisted of 0.06 M borate buffer (875 µL) and crude enzyme (250 µL). The reaction was initiated by the addition of L- phenylalanine (250 µL of 10 mg/mL^−1^, to give a final concentration of 11 mM). The tubes were incubated at 30 °C for 30 min and the reaction stopped by the addition of 35% *w*/*v* trifluoroacetic acid (125 µL). After, the tubes were then centrifuged for 5 min at 5000× *g* to pellet the denatured protein. PAL activity was estimated by measuring A_290_ of the supernatant in 10 mM quartz cuvettes, being determined from the yield of cinnamic acid. Triplicate assays were performed for each extract, both with and without a substrate to compensate for increases in absorbance in the absence of added phenylalanine [43].

### 4.8. Statistical Analysis

In the experiment, three replications were used and the results are expressed by means ±SD (standard deviation). To test the differences, a one-way ANOVA analysis was applied. Duncan’s multiple range test (DMRT) post hoc test specifies the differences between the categories as follows: healthy plants, and the three stages of the disease. A Pearson correlation showed the differences between secondary metabolites (TPC and TFC) and antioxidant activity (ABTS, DPPH, and FRAP). XLSTAT (2013) [44] was used for analyses.

## 5. Conclusions

The results of our study concentrated on physiological and biochemical changes that palm oil suffers during each stage of bud rot disease. Our resultsrevealed a gradually increasing implication of phenolic compounds and carotenoid synthesis, correlated with high antioxidant activity against ROS until the third stage of the disease. In this stage, the palm starts to decrease the antioxidative defense due to the high incidence of disease that provokes the palm to lose the fight against it, translated by its death initiation.

## Figures and Tables

**Table 1:** Total polyphenolic and flavonoid content in different stages of the disease.

	TPC (mg GAE/g DW)	TFC (mg QE/g DW)
S	21.619 ± 3.721 ^a^	0.178 ± 0.032 ^a^
E I	45.203 ± 1.162 ^b^	1.310 ± 0.108 ^b^
E II	33.397 ± 0.932 ^c^	0.730 ± 0.062 ^c^
E III	10.161 ± 0.628 ^d^	0.096 ± 0.018 ^a^

Legend: S—healthy palm oil; E I—stage I of bud rot disease infection in oil palm; E II—stage II of bud rot disease infection in oil palm; E III—stage III of bud rot disease infection in oil palm; TPC—total phenolic content; TFC—total flavonoid content. Values followed by different letters are significantly different on analyzed stages, and values followed by the same letter are not significant.

**Table 2:** Antioxidant capacity (ABTS, DPPH inhibition %, and reduction capacity–FRAP) during the different stages of the disease.

	DPPH	ABTS	FRAP
S	58.5 ± 0.14 ^a^	15.28 ± 0.056 ^a^	36.44 ± 0.02 ^a^
E I	75.981 ± 0.003 ^b^	42.582 ± 0.0085 ^b^	247.64 ± 0.03 ^b^
E II	69.042 ± 0.001 ^c^	15.07 ± 0.019 ^a^	144.86 ± 0.06 ^c^
E III	12.747 ± 0.01 ^d^	5.623 ± 0.007 ^c^	30.39 ± 0.0001 ^a^

Legend: S—healthy palm oil; E I—stage I of bud rot disease infection in oil palm; E II—stage II of bud rot disease infection in oil palm; E III—stage III of bud rot disease infection in oil palm. Values followed by different letters are significantly different on analyzed stages, and values followed by the same letter are not significant.

**Table 3:** Chlorophylls a, b, and carotenoids in different stages of the disease.

	Chlorophyll a (mg/g DW)	Chlorophyll b (mg/g DW)	Carotenoids (mg/g DW)	Chlorophyll a + b	Chlorophyll a + b/Carotenoids
S	4.9 ± 0.01	9.3 ± 0.128	2.49 ± 0.204	14.2 ± 0.125	5.703 ± 0.441
E I	3.7 ± 0.04	8.1 ± 0.115	3.02 ± 0.231	11.8 ± 0.147	3.907 ± 0.279
E II	2.4 ± 0.08	7.03 ± 0.026	3.49 ± 0.304	9.43 ± 0.079	2.702 ± 0.217
E III	1.8 ± 0.111	6.2 ± 0.815	3.1 ± 0.096	8 ± 0.139	2.581 ± 0.113

Legend: S–healthy palm oil; E I–stage I of bud rot disease infection in oil palm; E II–stage II of bud rot disease infection in oil palm; E III–stage III of bud rot disease infection in oil palm.

**Table 4:** PAL activity in different stages of the disease.

	PAL-Specific Activity (U/mg Protein)
S	1315.694 ± 71.108
E I	1771.667 ± 33.868
E II	1137.639 ± 57.010
E III	677.6389 ± 187.75

Legend: S—healthy palm oil; E I—stage I of bud rot disease infection in oil palm; E II—stage II of bud rot disease infection in oil palm; E III—stage III of bud rot disease infection in oil palm.

## Data Availability

Not applicable.

## Sample Availability

Samples of the compounds are not available from the authors.
